# Current Status, Challenges, and Future Directions in Crohn’s Disease

**DOI:** 10.3390/jcm13164699

**Published:** 2024-08-10

**Authors:** Christian Selinger, Andrea van der Meulen

**Affiliations:** 1 Leeds Gastroenterology Institute, Leeds Teaching Hospitals NHS Trust, St James University Hospital, 4th Floor Bexley Wing, Beckett Street, Leeds LS9 7TF, UK; 2 Department of Gastroenterology, Leiden University Medical Center, 2300 RC Leiden, The Netherlands; ae.meulen@lumc.nl

The treatment goal for patients with Crohn’s disease (CD) has traditionally been aimed at symptomatic steroid-free clinical remission [1]. Mucosal healing, endoscopic healing, and the prevention of exacerbation during maintenance therapy (with the aim of reducing the subsequent occurrence of complications, such as strictures, fistulas, and colorectal cancer) were proposed by the STRIDE-2 consensus [2]. Despite the increasing number of active drugs that can be used to treat IBD, treatment results remain disappointing for subsets of patients who continue to experience reduced quality of life [3]. Additionally, side effects may hamper optimal treatment with these drugs in some patients. Whether the STRIDE-2 recommendations are sufficiently evidence based and can be introduced to routine clinical practice remains controversial [4,5]. The following Special Issue provides an update on the current status, challenges, and future directions in Crohn’s disease.

Early diagnosis is often crucial for the prevention of complicated Crohn’s disease [6]. Scheurlen et al. (Contribution 1) highlight the challenges in making an early diagnosis; however, they note exciting recent developments in the field. Studies examining pre-clinical states of IBD have provided fascinating insights that may, in turn, revolutionise how we diagnose IBD. It is difficult, however, to accurately predict which patients are at risk of an aggressive disease course, and the results of the recent PROFILE study demonstrated that the PredictSURE blood test was not able to accurately predict disease course [7]. Furthermore, the authors highlight the current unmet needs of patients living with Crohn’s disease. 

The challenges of managing perianal Crohn’s disease are highlighted by Anandabaskaran et al. in their study (Contribution 2). In their review article, they describe how the recently adopted perianal CD classification allows for better categorisation of fistulas and clear treatment plans according to fistula type. Furthermore, they highlight the current evidence for medical and surgical treatment approaches. They note that a thorough description of the pathophysiology leads to a science-based assessment of how mesenchymal stem cell therapies, JAK inhibition, and ustekinumab may further improve the care of patients with perianal CD. 

While cryptoglandular [8] and CD-associated fistulising perianal disease may appear macroscopically similar, differentiation is crucial on a clinical level. In their study, Zhou et al. (Contribution 3) highlight differences in their complexity, treatment options, and healing rate. While cryptoglandular fistulas develop from anal gland inflammation, perianal CD fistulas arise from complex intestinal inflammation. The review authors highlight surgery as the cornerstone of cryptoglandular fistula treatment and how intervention for complex cryptoglandular disease including advancement flaps, fibrin glue, anal fistula plug (AFP), video-assisted anal fistula treatment (VAAFT), and fistula laser closure (FiLaC) all have reasonable success rates. In contrast, CD-associated fistulising perianal disease requires a combined medical–surgical approach, and the complex surgical interventions described above are usually associated with disappointingly low success rates. 

Kamal et al. (Contribution 4) argue in their study that the complexity and heterogeneity of Crohn’s disease are not currently reflected in conventional classification systems. The Montreal classification describes the phenotype but does not allow for the prediction of disease course [9]. Novel approaches that aim to include serological markers and multi-omics approaches including genomics, transcriptomics, proteomics, epigenomics, metagenomics, metabolomics, lipidomics, and immunophenomics may, in the future, allow us to classify Crohn’s disease in a manner that can be helpful for treatment decisions and disease course prediction. 

Fibrosis is a common phenomenon in healing intestinal tissue and can lead to symptomatic stricturing of Crohn’s disease. Thus far, there are no clinically meaningful predictors of strictures nor are there available treatment modalities to combat fibrosis. The advent of single-cell sequencing has led to significant advances in our understanding of the disease at the cellular level. Campbell et al. (Contribution 5) review the current understanding of the pathophysiology of fibrosis in Crohn’s disease. They summarise current treatments and highlight how single-cell sequencing may be the key to developing effective therapies. 

Many women with Crohn’s disease are of childbearing age, and management of pregnancies in these women is often complex. While the management of IBD during pregnancy is often discussed in the literature, in their review article, Rosiou and Selinger (Contribution 6) focus on the management of obstetric considerations in pregnant women with Crohn’s disease. Discussions of fertility in the disease course during pregnancy are followed by advice on risk factors for adverse maternal and fetal outcomes. The focus on delivery allows clinicians to advise their patients on the optimal mode of delivery. 

Around the globe, there is an increasing prevalence of IBD, and in developed countries, this increase is predominantly driven by the long life expectancy of patients with IBD. Hence, we are seeing an increasing number of patients over 60 years of age in our IBD clinics. The risk of adverse health outcomes often potentially related to IBD treatment correlates better with biological than with chronological age. Fons et al. (Contribution 7) review frailty in IBD patients. They define frailty, examine its prevalence in IBD patients, and describe the outcomes associated with frailty. It is paramount that we shift away from basing treatment decisions on chronological age and instead focus on frailty. We should not deny fit older patients treatment based on their chronological age alone. 

Crises can often prompt great innovations and changes in approach. The COVID-19 pandemic was certainly one of the biggest global crises to affect healthcare. Zhang et al. (Contribution 8) review the effects of the pandemic on patients with IBD. They review the risk of COVID-19 infection, the response to COVID-19 vaccines, and the treatment of COVID-19 in patients diagnosed with IBD. In addition, they reflect on positive changes brought about or at least accelerated by the pandemic. Telemedicine has provided patients with greater choice, reduced carbon emissions, and is generally favored by patients. Non-invasive monitoring with faecal calprotectin and bowel ultrasound can replace more invasive endoscopies in many scenarios. Point-of-care testing of faecal calprotectin in the patient’s home may reduce the need for travel. Finally, subcutaneous versions of established biologics such as infliximab and vedolizumab allow for a reduction in travel and time spent on healthcare for patients. 

Radiological imaging remains crucial in the assessment of small bowel or perianal Crohn’s disease. While magnetic resonance imaging (MRI) offers a radiation-free method, computed tomography (CT) is an often-used radiation-exposure method. Yang et al. (Contribution 9) examine radiation exposure in a single-center cohort. Unsurprisingly, the rate of radiation exposure was higher for patients with Crohn’s disease than for those diagnosed with ulcerative colitis. Nearly 7% of patients with Crohn’s disease were classed as having experienced high radiation exposure. Therefore, clinicians need to focus on using MRI or small bowel ultrasound over CT and reserve the latter for emergency situations. 

Anti-TNF medications are often recommended as a first-line choice of advanced therapy for Crohn’s disease [3]. However, many patients experience a loss of response [10], which necessitates second-line advanced therapies. Sharip et al. (Contribution 10) examine studies containing real-world evidence by examining the effectiveness of ustekinumab versus vedolizumab in this setting. They conclude that most studies find ustekinumab to be superior or at least non-inferior to vedolizumab, though many confounders remain despite best efforts by researchers to adjust for these factors. Lastly, the increasing use of JAK inhibitors in Crohn’s disease patients highlights the need for further studies on second-line therapy. 

In summary, the future for patients with Crohn’s disease seems brighter due to increased choice of effective medication, new approaches to classifying disease and assessing disease effectively and safely, and the appropriate management of Crohn’s disease through the different life phases of our patients.

**List of Contributions**

1. Scheurlen, K.M.; A Parks, M.; Macleod, A.; Galandiuk, S. Unmet Challenges in Patients with Crohn’s Disease. *J. Clin. Med.* **2023**, *12*, 5595.
2. Anandabaskaran, S.; Hanna, L.; Iqbal, N.; Constable, L.; Tozer, P.; Hart, A. Where Are We and Where to Next?-The Future of Perianal Crohn’s Disease Management. *J. Clin. Med.* **2023**, *12*, 6379.
3. Zhou, Z.; Ouboter, L.F.; Peeters, K.C.; Hawinkels, L.J.; Holman, F.; Pascutti, M.F.; Barnhoorn, M.C.; van der Meulen-de Jong, A.E. Crohn’s Disease-Associated and Cryptoglandular Fistulas: Differences and Similarities. *J. Clin. Med.* **2023**, *12*, 466.
4. Kamal, S.; Parkash, N.; Beattie, W.; Christensen, B.; Segal, J.P. Are We Ready to Reclassify Crohn’s Disease Using Molecular Classification? *J. Clin. Med.* **2023**, *12*, 5786.
5. Campbell, I.; Glinka, M.; Shaban, F.; Kirkwood, K.J.; Nadalin, F.; Adams, D.; Papatheodorou, I.; Burger, A.; Baldock, R.A.; Arends, M.J.; et al. The Promise of Single-Cell RNA Sequencing to Redefine the Understanding of Crohn’s Disease Fibrosis Mechanisms. *J. Clin. Med.* **2023**, *12*, 3884.
6. Rosiou, K.; Selinger, C.P. Obstetric Considerations in Pregnant Women with Crohn’s Disease. *J. Clin. Med.* **2023**, *12*, 684.
7. Fons, A.; Kalisvaart, K.; Maljaars, J. Frailty and Inflammatory Bowel Disease: A Scoping Review of Current Evidence. *J. Clin. Med.* **2023**, *12*, 533.
8. Zhang, E.; Christensen, B.; Macrae, F.A.; Leong, R. The Effects of the COVID Pandemic on Patients with IBD: Lessons Learned and Future Directions. *J. Clin. Med.* **2022**, *11*, 7002.
9. Yang, C.T.; Yen, H.H.; Chen, Y.Y.; Su, P.Y.; Huang, S.P. Radiation Exposure among Patients with Inflammatory Bowel Disease: A Single-Medical-Center Retrospective Analysis in Taiwan. *J. Clin. Med.* **2022**, *11*, 5050.
10. Sharip, M.T.; Nishad, N.; Pillay, L.; Goordyal, N.; Goerge, S.; Subramanian, S. Ustekinumab or Vedolizumab after Failure of Anti-TNF Agents in Crohn’s Disease: A Review of Comparative Effectiveness Studies. *J. Clin. Med.* **2024**, *13*, 2187.
